# Polyphyllin VI Induces Caspase-1-Mediated Pyroptosis via the Induction of ROS/NF-κB/NLRP3/GSDMD Signal Axis in Non-Small Cell Lung Cancer

**DOI:** 10.3390/cancers12010193

**Published:** 2020-01-13

**Authors:** Jin-Feng Teng, Qi-Bing Mei, Xiao-Gang Zhou, Yong Tang, Rui Xiong, Wen-Qiao Qiu, Rong Pan, Betty Yuen-Kwan Law, Vincent Kam-Wai Wong, Chong-Lin Yu, Han-An Long, Xiu-Li Xiao, Feng Zhang, Jian-Ming Wu, Da-Lian Qin, An-Guo Wu

**Affiliations:** 1 Sichuan Key Medical Laboratory of New Drug Discovery and Drugability Evaluation, Luzhou Key Laboratory of Activity Screening and Druggability Evaluation for Chinese Materia Medica, School of Pharmacy, Southwest Medical University, Luzhou 646000, China; 18883178848@163.com (J.-F.T.); qbmei@swmu.edu.cn (Q.-B.M.); zxg@swmu.edu.cn (X.-G.Z.); tangy1989@yeah.net (Y.T.); rxiong2017@sina.com (R.X.); fanny95qiu@163.com (W.-Q.Q.); zhangf037@163.com (F.Z.); dalianqin@swmu.edu.cn (D.-L.Q.); 2 Department of Human Anatomy, School of Preclinical Medicine, Southwest Medical University, Luzhou 646000, China; pr18982779805@163.com (R.P.); 8056ycl@swmu.edu.cn (C.-L.Y.); 3 State Key Laboratory of Quality Research in Chinese Medicine, Macau University of Science and Technology, Macau 999078, China; lykbetty@gmail.com (B.Y.-K.L.); bowaiwong@gmail.com (V.K.-W.W.); 4 Education Ministry Key Laboratory of Medical Electrophysiology, Southwest Medical University, Luzhou 646000, China

**Keywords:** NSCLC, polyphyllin VI, pyroptosis, ROS/NF-κB/NLRP3/GSDMD

## Abstract

*Trillium tschonoskii* Maxim (TTM), a traditional Chinese medicine, has been demonstrated to have a potent anti-tumor effect. Recently, polyphyllin VI (PPVI), a main saponin isolated from TTM, was reported by us to significantly suppress the proliferation of non-small cell lung cancer (NSCLC) via the induction of apoptosis and autophagy in vitro and in vivo. In this study, we further found that the NLRP3 inflammasome was activated in PPVI administrated A549-bearing athymic nude mice. As is known to us, pyroptosis is an inflammatory form of caspase-1-dependent programmed cell death that plays an important role in cancer. By using A549 and H1299 cells, the in vitro effect and action mechanism by which PPVI induces activation of the NLRP3 inflammasome in NSCLC were investigated. The anti-proliferative effect of PPVI in A549 and H1299 cells was firstly measured and validated by MTT assay. The activation of the NLRP3 inflammasome was detected by using Hoechst33324/PI staining, flow cytometry analysis and real-time live cell imaging methods. We found that PPVI significantly increased the percentage of cells with PI signal in A549 and H1299, and the dynamic change in cell morphology and the process of cell death of A549 cells indicated that PPVI induced an apoptosis-to-pyroptosis switch, and, ultimately, lytic cell death. In addition, belnacasan (VX-765), an inhibitor of caspase-1, could remarkably decrease the pyroptotic cell death of PPVI-treated A549 and H1299 cells. Moreover, by detecting the expression of NLRP3, ASC, caspase-1, IL-1β, IL-18 and GSDMD in A549 and h1299 cells using Western blotting, immunofluorescence imaging and flow cytometric analysis, measuring the caspase-1 activity using colorimetric assay, and quantifying the cytokines level of IL-1β and IL-18 using ELISA, the NLRP3 inflammasome was found to be activated in a dose manner, while VX-765 and necrosulfonamide (NSA), an inhibitor of GSDMD, could inhibit PPVI-induced activation of the NLRP3 inflammasome. Furthermore, the mechanism study found that PPVI could activate the NF-κB signaling pathway via increasing reactive oxygen species (ROS) levels in A549 and H1299 cells, and *N*-acetyl-L-cysteine (NAC), a scavenger of ROS, remarkably inhibited the cell death, and the activation of NF-κB and the NLRP3 inflammasome in PPVI-treated A549 and H1299 cells. Taken together, these data suggested that PPVI-induced, caspase-1-mediated pyroptosis via the induction of the ROS/NF-κB/NLRP3/GSDMD signal axis in NSCLC, which further clarified the mechanism of PPVI in the inhibition of NSCLC, and thereby provided a possibility for PPVI to serve as a novel therapeutic agent for NSCLC in the future.

## 1. Introduction

Lung cancer, as one of the most common malignant tumors, is the leading cause of cancer-related death worldwide [1]. It has a dismal prognosis and a five year survival rate lower than 15% [2]. Non-small cell lung cancer (NSCLC), which includes squamous cell carinoma, adenocarcinoma and large-cell carcinoma, is a classic subtype of lung cancer and accounts for 85% of all lung cancer cases [3]. Although there are many therapeutic methods, including surgery, radiotherapy and chemotherapy, chemotherapy is still the common strategy for lung cancer treatment [4,5]. However, chemotherapy is less sensitive to NSCLC as compared to small lung cancer [6]. Therefore, novel strategies are essential for the improvement of clinical survival rate based on a better understanding of tumor biology. 

Pyroptosis, also known as inflammatory necrosis, is a highly inflammatory form of programmed cell death, which was first discovered in 2001 by Dr. Brad T. Cookson [7], and is characterized by cell swelling and large bubbles emerging from the plasma membrane [8,9]. Recently, emerging evidence shows that pyroptosis plays a critical role in inhibiting the proliferation of tumor cells in vitro and tumor growth in vivo [10]. Pyroptosis has a distinct morphology and mechanism as compared to other types of cell death [11,12]. Caspase-1, an interleukin-1 converting enzyme (ICE), proteolytically cleaves the precursors of the inflammatory cytokines, such as interleukin-1β (IL-1β) and IL-18, as well as the pyropotsis inducer Gasdermin D (GSDMD), into mature and active forms [12]. The pro-capsase-1 can be recruited by various inflammasomes and is activated within the inflammasome following its assembly [13]. NACHT, LRR and PYD domains-containing protein 3 (NLRP3), also known as NALP3 and cryopyrin, belonging to the NOD-like receptor (NLR), together with the adaptor apoptosis-associated speck-like protein containing a CARD (ASC) protein PYCARD, a N-terminal PYRIN-PAAD-DAPIN domain (PYD) and a C-terminal caspase-recruitment domain (CARD), to form a caspase-1 activating complex known as the NLRP3 inflammasome [14]. Increasing evidence indicates that the NLRP3 inflammasome, the most characterized and studied inflammasome, responds to various activators, such as microorganisms and their derived products, as well as endogenous danger signals [15,16,17]. Activation of the NLRP3 inflammasome requires the following two distinct signals: the first is to synthesize the nuclear factor kappa B (NF-κB)-mediated NLRP3, and pro-IL-1β and IL-18 expression by inflammatory stimuli such as TLR4 agonists; the second signal is the assembly of the NLRP3 inflammasome, caspase-1 activation, and IL-1β and IL-18 secretions [14,18]. Oxidative stress is characterized by an imbalance between the production and accumulation of reactive oxygen species (ROS), which are mainly from the damaged mitochondria [19]. Emerging evidences indicate that ROS, as second messengers in the physiology of cells, play an important role in controlling tumor growth and signaling transduction [20]. Recently, ROS has also been considered to be one of the first identified triggers of NLRP3 inflammasome activation [21], and many chemical compounds were reported to activate NLRP3 inflammasome via increasing intracellular ROS levels [22]. Meanwhile, N-acetyl and P22 (phox), the typical ROS scavengers, could significantly downregulate the NLRP3 inflammasome [23]. Therefore, ROS generation is essential for the activation of the NLRP3 inflammasome. In addition, ROS also plays a critical role in the signal transduction pathways. The NF-κB pathway was initially thought to regulate inflammasome activation and improve NLRP3 protein expression [21]. Meanwhile, many studies showed that *N*-acetyl-L-cysteine (NAC) could inhibit the NF-κB signaling pathway, and thus suppress the transcription in vivo and in vitro, resulting in a decrease in pro-inflammatory cytokines secretion [24]. Therefore, the ROS-mediated NF-κB signaling pathway plays an important role in the activation of the NLRP3 inflammasome.

To date, many approved drugs with potent anti-tumor effects, such as vinca alkaloids, podophyllotoxins, taxanes, camptothecins, etc., were isolated from the natural plants. Traditional Chinese medicines (TCMs) in China have a 2000 year history and have proven to be safe and effective in the prevention and treatment of diverse diseases [25]. *Trillium tschonoskii* Maxim. (TTM), also known as Yan Ling Cao in Chinese, a folk medical herb that is commonly used in China, has many pharmacological effects, such as blood pressure reduction, neuroprotection, anti-inflammatory, analgesia and hemolysis, and anti-aging [26,27]. Furthermore, we have previously reported that TTM possessed potent anti-tumor effects in cell and animal models [28]. Moreover, polyphyllin VI (PPVI), a main saponin in TTM, was previously reported by us to significantly suppress NSCLC in vitro and in vivo. In this study, the NLRP3 inflammasome was found to be activated in PPVI-administrated, A549-bearing athymic nude mice; the further study revealed that PPVI induced an apoptosis-to-pyroptosis switch and ultimately cell death in A549 and H1299 cells via the activation of caspase-1. In addition, PPVI-induced activation of the NLRP3 inflammasome was closely associated with the ROS/NF-κB/NLRP3/GSDMD signal axis. Therefore, this study clarified the mechanism of PPVI in the inhibition of NSCLC for the first time, and demonstrated that PPVI is valuable for the further development of a new candidate for the treatment of NSCLC in the future.

## 2. Results

### 2.1. PPVI Activates NLRP3 Inflammasome in A549-Bearing Athymic Nude Mice

**Figure 1:**
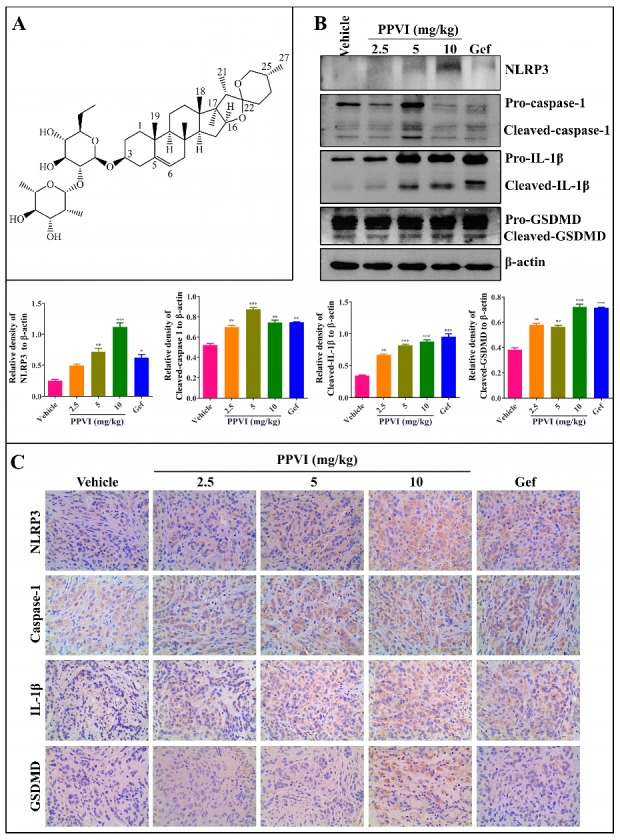
Polyphyllin VI (PPVI) activates the NLRP3 inflammasome in A549 bearing athymic nude mice. (**A**) Chemical structure of PPVI. (**B**) Tumor tissue lysates were analyzed by Western blot for NLRP3, caspase-1, IL-1β, GSDMD and β-actin. Bar chart indicates the relative density of the protein to β-actin; bars, S.D. ** *p* ≤ 0.01; *** *p* ≤ 0.001. The full-length Western blotting images are shown in Figure S4. (**C**) The expression of NLRP3, caspase-1, IL-1β and GSDMD in the tumor tissue of A549-bearing athymic nude mice were analyzed by the immunohistochemistry method. Magnification: 40×, Scale bar: 40 µm.

The PPVI shown in Figure 1A, a main saponin in TTM, has been previously demonstrated by us to significantly inhibit the proliferation of NSCLC via the ROS-triggered, mTOR-mediated apoptotic and autophagic cell death in vitro and in vivo [29]. Recently, emerging evidences indicate that pyroptosis also plays an important role in cancer [30]. Through further detection of the NLRP3 inflammasome in the tumor tissue of A549-bearing athymic nude mice using Western blotting and immunohistochemistry methods, Figure 1B showed that PPVI significantly improved the protein expression of NLRP3, cleaved-caspase-1, cleaved-IL-1β and cleaved-GSDMD in tumor tissue. Furthermore, the immunohistochemistry results in Figure 1C showed that PPVI significantly increased the expression of NLRP3, caspase-1, IL-1β and GSDMD in a dose manner. Taken together, the present in vivo experiment suggests that PPVI could activate the NLRP3 inflammasome in A549-bearing athymic nude mice.

### 2.2. PPVI Induces Distinct Patterns of Apoptosis and Lytic Cell Death in A549 and H1299 Cells

**Figure 2:**
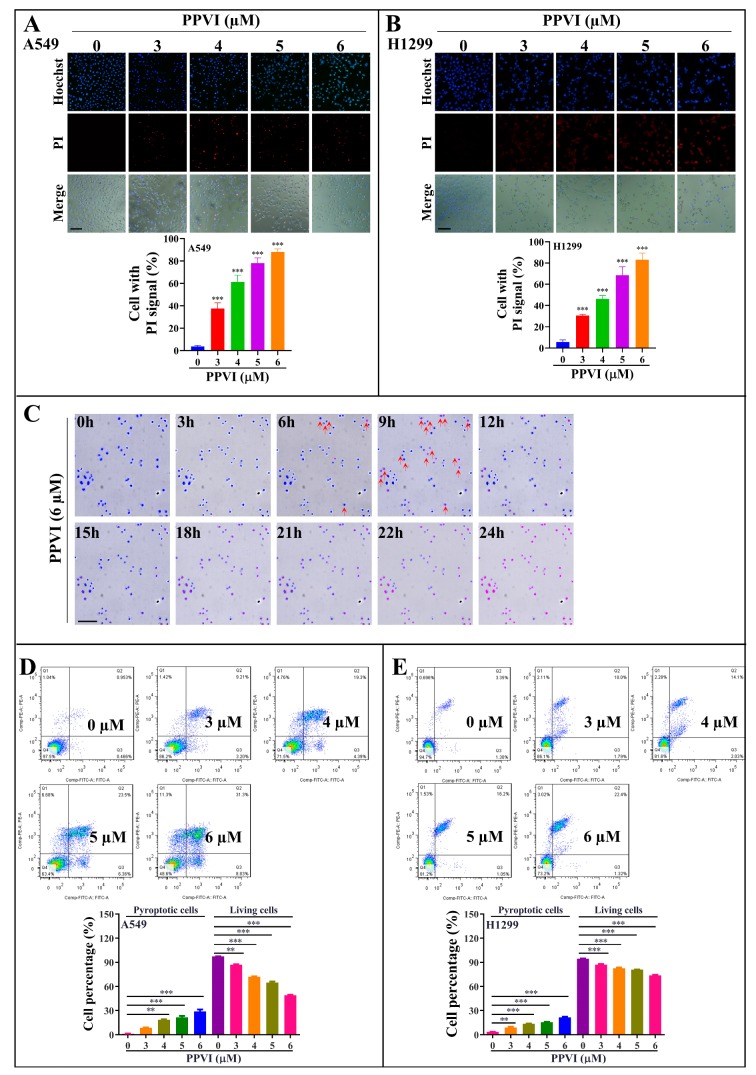
PPVI induces lytic cell death in A549 and H1299 cells. A549 cells (**A**) and H1299 cells (**B**) were treated with PPVI for 24 h. The cells were then stained with 2 μg/mL PI (red, staining dying cells) plus 2 μg/mL Hoechst 33342 (blue, staining all cells) for 10 min, and observed by fluorescent microscopy. Bar chart indicates the percentage of cells with PI signal to blue signal; bars, S.D. *** *p* ≤ 0.001. Magnification: 10×, Scale bar: 25 µm. (**C**) The time-lapse phase-contrast and fluorescent images of A549 cells stained with PI and Hoechst 33342 solution were taken at the indicated timepoints after the stimulation of 6 μM PPVI. The images in the red, blue and white channels were merged at the same field. Red arrow indicated the cells at 6–9 h, beginning with membrane blebbing and producing apoptotic body-like cell protrusions (termed pyroptotic bodies) prior to plasma membrane rupture. The real-time video was included in Video S1. Data shown are representative of at least three independent experiments. Magnification: 10×, Scale bar: 100 µm. Pyroptotic cells or living cells of A549 cells (**D**) and H1299 cells (**E**) treated as indicated graded concentrations were measured by flow cytometry using an annexin V-FITC/PI apoptotic detecting kit. Annexin V^+^/PI^+^ indicated the pyroptotic cells and annexin-V^−^/PI^−^ represented living cells. Bar chart indicates the percentage of pyroptotic cells and living cells; bars, S.D. ** *p* ≤ 0.01, *** *p* ≤ 0.001.

In this study, the anti-proliferative effect of PPVI at 24, 48 and 72 h timepoints was firstly investigated and confirmed in A549 and H1299 cells, which was consistent with our previously reported result (Figure S1A,B) [29]. Furthermore, the MTT result indicated that PPVI exhibited a similar inhibitive effect among the wild type (WT) EGFR NSCLC cell lines (A549 and H1299) and mutated-EGFR cell line (PC-9) (Figure S1C). To reveal the type of cell death induced by PPVI, A549 and H1299 cells were doubly stained with Hoechst33324/PI, the nuclei of cells was stained by Hoechst33324, while PI could penetrate into the dying cells with the loss of cell membrane integrity. As shown in Figure 2A,B, the Hoechst33324 staining displayed that the cell density of A549 or H1299 cells was decreased by PPVI, and PI staining indicated that the proportion of PPVI-induced lytic cells death (PI-positive cells) was increased in a dose manner. Furthermore, the dynamic change in cell morphology and the cell death process of A549 cells were observed by an ImageXpress Micro 4 Widefield High-Content Imaging System. A total of 6 μM of PPVI-induced A549 cells gradually shrank and the PI uptake into cells increased, followed by the swell and rupture of the cell membrane, and, ultimately, cell death (Figure 2C and Video S1). These imaging results clearly indicate that PPVI-induced A549 cells underwent an apoptosis-to-pyroptosis switch, and, ultimately, lytic cell death. Moreover, we also used annexin V-FITC/PI together with flow cytometry to analyze the percentage of cell viability, early apoptosis, late apoptosis, and the pyroptosis of A549 and H1299 cells after being treated by PPVI for 24 h. Similar concentrations of PPVI were used and the results showed that PPVI significantly and dose-dependently increased the rate of pyroptotic cells, and correspondingly decreased the cell viability of A549 and H1299 cells (Figure 2D,E) [31]. Taken together, the above results suggested that PPVI significantly induced lytic cell death (pyroptosis) in A549 and H1299 cells.

### 2.3. PPVI Induces Cell Death via the Activation of Caspase-1 in A549 and H1299 Cells

**Figure 3:**
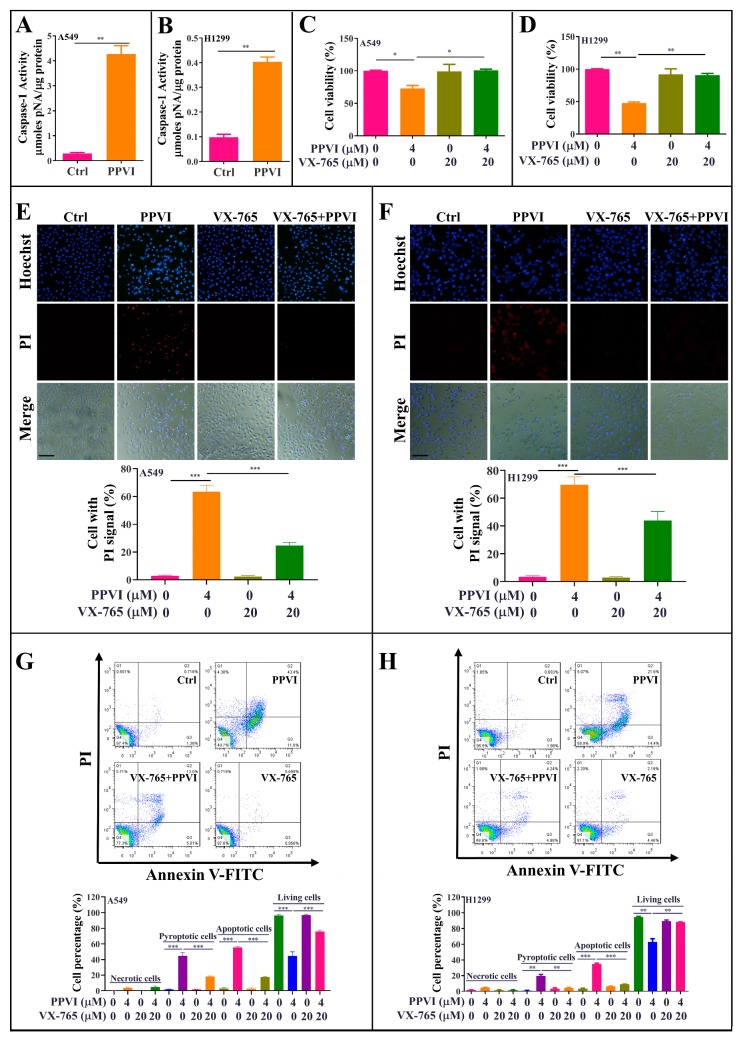
PPVI induces cell death via the activation of caspase-1 in A549 and H1299 cells. A549 cells (**A**) and H1299 cells (**B**) were treated with 4 μM PPVI for 24 h; the activated caspase-1 were then measured by using a Caspase-1 Activity Assay Kit (Colorimetric) according to the manufacturer’s instructions. Bar chart indicated the amount of pNA in A549 and H1299 cells. A549 cells (**C**) and H1299 cells (**D**) were treated with PPVI or co-treated with PPVI and VX-765 at the indicated concentration for 24 h. After treatment, the cytotoxicity was measured by using MTT assay. Bar chart indicates the cell viability of A549 and H1299 cells; bars, S.D. * *p* ≤ 0.05; ** *p* ≤ 0.01. A549 cells (**E**) and H1299 cells (**F**) were treated with PPVI or co-treated with PPVI and VX-765 at the indicated concentration for 24 h. The cells were then stained with 2 μg/mL PI (red, staining dying cells) plus 2 μg/mL Hoechst 33342 (blue, staining all cells) for 10 min, and observed by fluorescent microscopy. Bar chart indicates the percentage of cells with PI signal compared to blue signal. Magnification: 10×, Scale bar: 100 µm. Necrotic cells, pyroptotic cells, apoptotic cells or living cells of A549 cells (**G**) and H1299 cells (**H**), treated as indicated graded concentrations, were measured by flow cytometry using an annexin V-FITC/PI apoptotic detecting kit. Annexin V^-^/PI^+^ indicated the necrotic cells, annexin V^+^/PI^+^ indicated the pyroptotic cells, annexin V^+^/PI^−^ represented apoptotic cells and annexin-V^−^/PI^−^ represented living cells. Bar chart indicates the percentage of necrotic cells, pyroptotic cells, apoptotic cells and living cells; bars, S.D. ** *p* ≤ 0.01, *** *p* ≤ 0.001.

Emerging evidences indicate that caspase-1 can proteolytically cleave the precursors of the inflammatory cytokines such as interleukin-1β (IL-1β) and IL-18, as well as the GSDMD, into mature and active forms, and ultimately initiates cell pyroptotic death [32]. In this study, to investigate whether PPVI-induced pyroptotic death in A549 and H1299 cells links with the cleavage of caspase-1, we employed a Caspase-1 Activity Assay Kit (Colorimetric) to detect the activated caspase-1. As shown in Figure 3A,B, 4 μM PPVI significantly increased the level of activated caspase-1 in A549 and H1299 cells. In addition, VX-765, a specific inhibitor of caspase-1, was also employed. The MTT result shown in Figure 3A,B indicated that VX-765 significantly improved the cell viability of PPVI-treated A549 and H1299 cells. In the meantime, the LIVE/DEAD cell imaging results also displayed that the percentage of cell with red fluorescence in PPVI-treated A549 and H1299 cells was remarkably decreased by VX-765 (Figure S2). Moreover, the Hoechst33324/PI staining (Figure 3C,D) and flow cytometry analysis (Figure 3E,F) results showed that VX-765 could significantly decrease the proportion of PPVI-induced cells with PI uptake and the percentage of pyroptotic cells in A549 and H1299 cells, respectively, and ultimately reduced the cell death of A549 and H1299 cells. Taken together, these data suggest that PPVI induced cell pyroptotic death via the activation of caspase-1 in A549 and H1299 cells.

### 2.4. PPVI Activates NLRP3 Inflammasome in A549 and H1299 Cells

**Figure 4:**
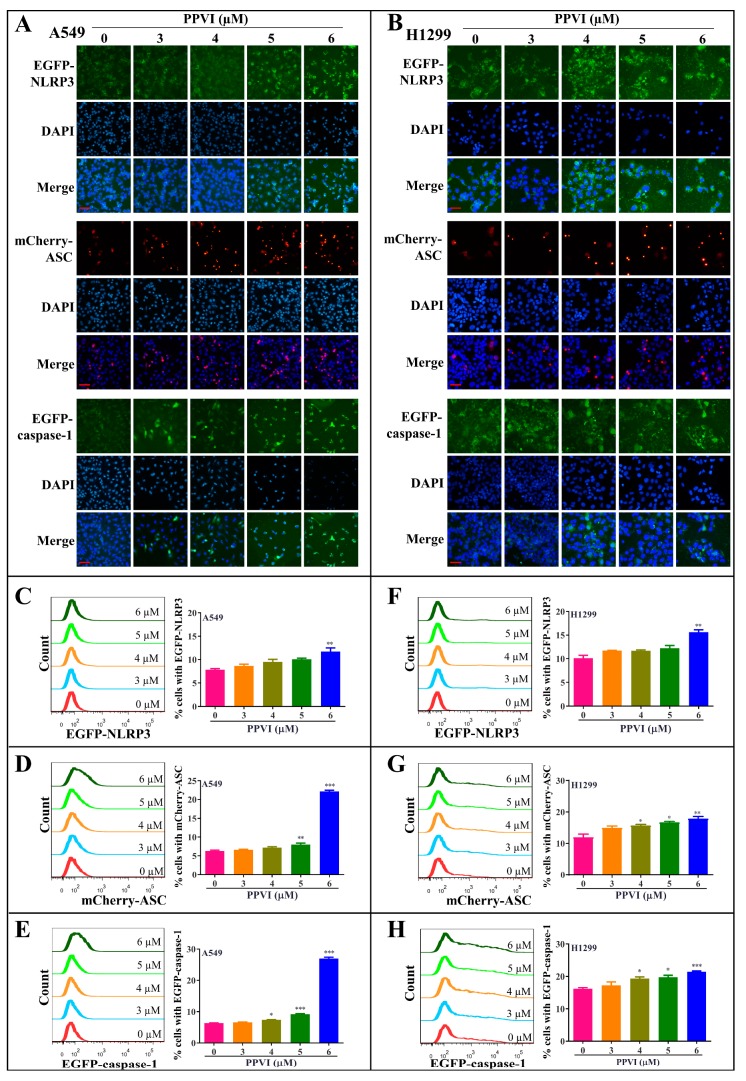
PPVI increased the expression of NLRP3, ASC and caspase-1 in A549 and H1299 cells. A549 cells (**A**) and H1299 cells (**B**) transiently transfected with EGFP-N1-NLRP3, mCherry-C1-ASC and EGFP-N1-caspase-1 plasmids for 24 h were treated with PPVI as indicated by the graded concentrations for another 24 h. After treatment, the cells were fixed with 4% PFA, and the representative images were captured using the fluorescence microscope. Magnification: 40×. Scale bar: 25 μm. A549 cells (**C**–**E**) and H1299 cells (**F**–**H**) transiently transfected with EGFP-N1-NLRP3, mCherry-C1-ASC and EGFP-N1-caspase-1 plasmids for 24 h were then treated with PPVI as indicated by the graded concentrations for another 24 h. After treatment, the cells were trypsinzed and collected for the analysis of fluorescence intensity by flow cytometry. Bar chart indicates the percentage of cells with GFP or mCherry signal in A549 and H1299 cells; bars, S.D. * *p* ≤ 0.05; ** *p* ≤ 0.01; *** *p* ≤ 0.001.

**Figure 5:**
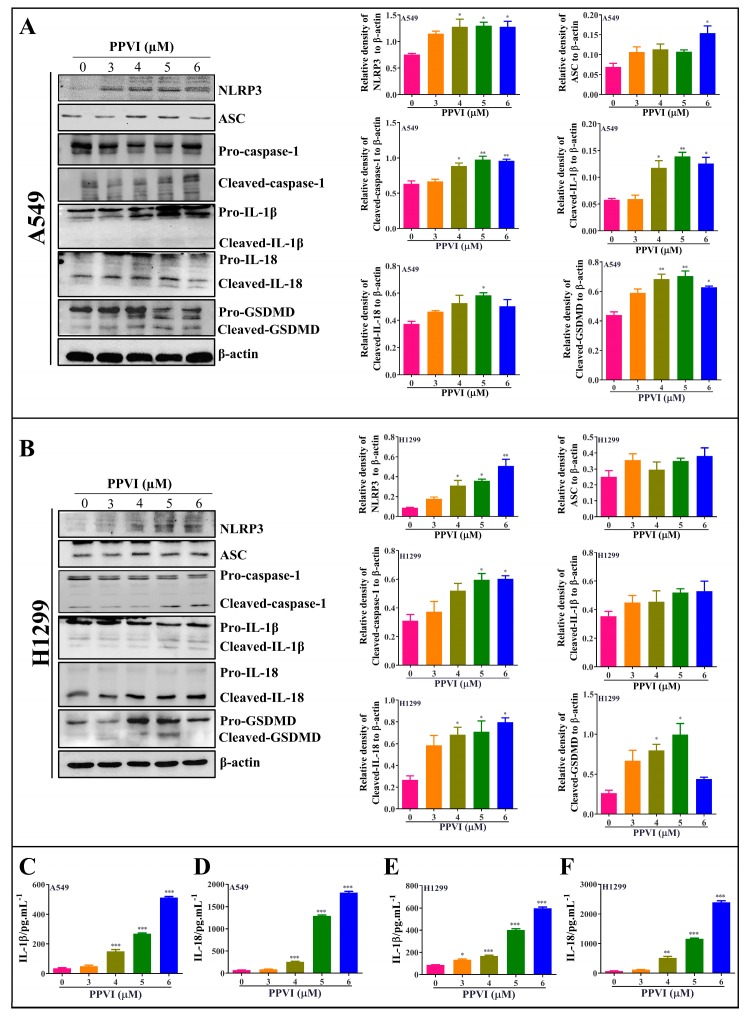
PPVI activated the NLRP3 inflammasome and promoted the release of IL-1β and IL-18. A549 cells (**A**) and H1299 cells (**B**) were treated with PPVI as indicated by the graded concentrations for 24 h. Cell protein was then harvested for detecting NLRP3, ASC, caspase-1, IL-1β, IL-18, GSDMD and β-actin by Western blot. Bar chart indicates the relative density of protein to β-actin; bars, S.D. * *p* ≤ 0.05; ** *p* ≤ 0.01. The full-length Western blotting images are shown in Figure S5. A549 cells and H1299 cells were treated with PPVI as indicated by gradient concentrations for 24 h. Culture medium was collected for centrifugation and the supernatant was used for the detection of the level of IL-1β and IL-18 by using a Human IL-1β ELISA Kit (CHE0001) and a Human IL-18 ELISA Kit (CHE0007). Bar chart indicates the level of IL-1β and IL-18 in the cell supernatant of A549 cells (**C**,**D**) and H1299 cells (**E**,**F**), respectively. bars, S.D. * *p* ≤ 0.05; ** *p* ≤ 0.01. *** *p* ≤ 0.001.

**Figure 6:**
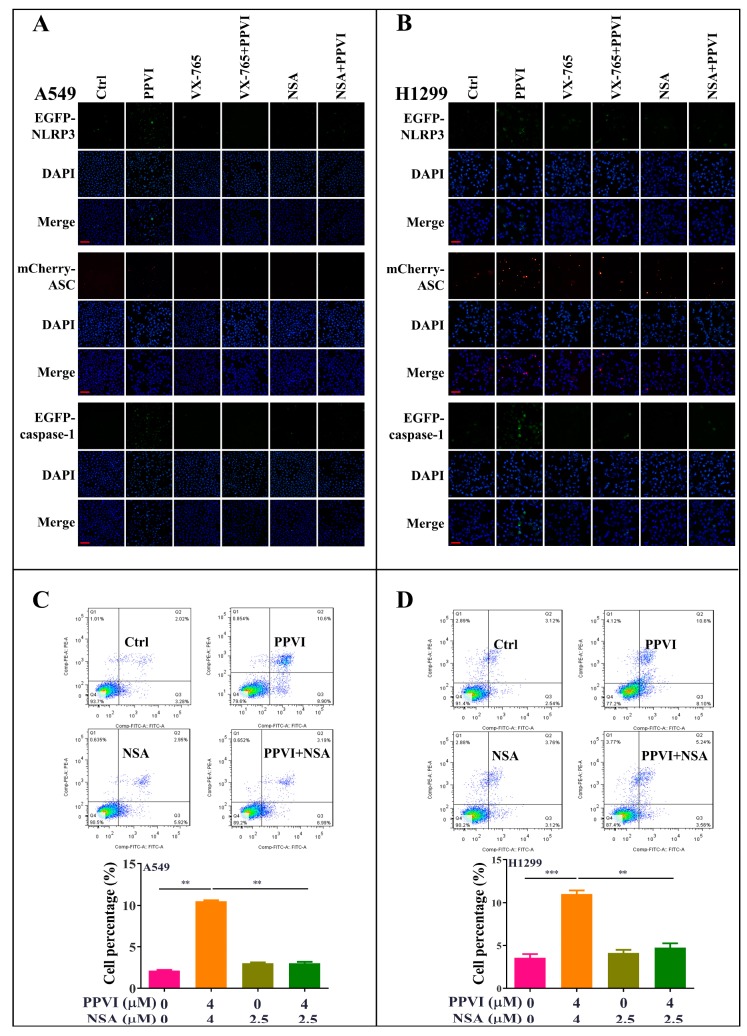
PPVI VX-765 and NSA reversed the activation of the NLRP3 inflammasome of PPVI-treated A549 and H1299 cells. A549 cells (**A**) and H1299 cells (**B**), transiently transfected with EGFP-N1-NLRP3, mCherry-C1-ASC and EGFP-N1-caspase-1 plasmids for 24 h, were treated with PPVI or co-treated with VX-765 or NSA and PPVI, as per the indicated concentration, for another 24 h. After treatment, the cells were fixed with 4% PFA, and the representative images were captured using the fluorescence microscope. Magnification: 20×. Scale bar: 50 μm. Pyroptotic cells of A549 cells (**C**) and H1299 cells (**D**) treated with PPVI or co-treated with NSA and PPVI, as indicated. Treatment concentrations were measured by flow cytometry using an annexin V-FITC/PI apoptotic detecting kit. Annexin V^+^/PI^+^ indicated the pyroptotic cells. Bar chart indicates the percentage of pyroptotic cells and living cells; bars, S.D. ** *p* ≤ 0.01, *** *p* ≤ 0.001.

It has been reported that the inactive zymogen form of caspase-1 autoactivates when it is assembled into the filamentous inflammasome complex [33]. The activation of the NLRP3 inflammasome, the most commonly studied inflammasome, in PPVI-treated A549 and H1299 cells, was investigated in this study. Firstly, A549 and H1299 cells transfected with EGFP-NLRP3, mCherry-ASC and EGFP-caspase-1 were treated with PPVI under the indicated concentrations for 24 h. After treatment, the captured representative images showed that PPVI significantly and dose-dependently increased EGFP-NLRP3 expression in A549 and H1299 cells. Consistent with this, we observed that PPVI could induce mCherry-ASC speck formation, which is required for the cleavage of caspase-1. Therefore, the expression of EGFP-caspase-1 was also found to be increased in PPVI-treated A549 and H1299 cells, respectively (Figure 4A,B). In addition, the intensity of GFP and RFP, representing the expression of NLRP3, caspase-1 or ASC, was detected and quantitated using flow cytometry. As shown in Figure 4C–E, PPVI significantly increased the cell percentage with GFP-NLRP3, mCherry-ASC and EGFP-caspase-1 in a dose-dependent manner, which was consistent with the results in H1299 cells (Figure 4F–H). Furthermore, the protein expression of NLRP3, ASC, caspase-1 and the pro-inflammatory cytokines, such as IL-1β and IL-18, and GSDMD, was detected by Western blot. As shown in Figure 5A,B, PPVI dose-dependently increased the protein expression of NLRP3 and ASC, and the cleaved form of caspase-1, IL-1β, IL-18 and GSDMD, which was accompanied by the gradient-increased secretion of IL-1β and IL-18 into the cell supernatant of A549 cells (Figure 5C,D) and H1299 cells (Figure 5E,F). Moreover, VX-765 and NSA (a specific inhibitor of GSDMD) could significantly decrease PPVI-induced expression of NLRP3, ASC and caspase-1 in transiently transfected EGFP-NLRP3, mCherry-ASC and EGFP-caspase-1 A549 and H1299 cells (Figure 6A,B). In addition, the flow cytometry analysis result shown in Figure 6C,D suggested that NSA significantly decreased the percentage of pyroptotic cells in Annexin V-FITC/PI-stained A549 and H1299 cells. Taken together, these data indicate that PPVI could activate the NLRP3 inflammasome, and then promote the release level of inflammatory cytokines and the cleavage of GSDMD in A549 and H1299 cells.

### 2.5. PPVI Increases the Intracellular ROS Level in A549 and H1299 Cells

**Figure 7:**
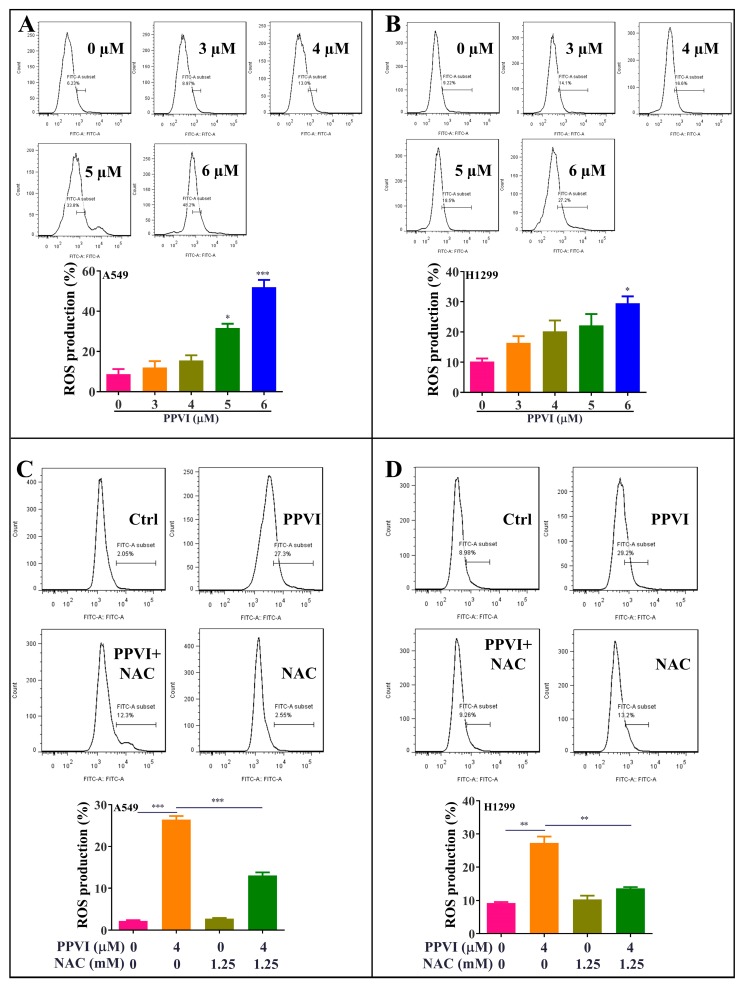
PPVI increased the intracellular reactive oxygen species (ROS) levels in A549 and H1299 cells. A549 cells (**A**) and H1299 cells (**B**) were treated with PPVI as indicated graded by the concentrations for 24 h. The cells were then incubated with 5 µM H2DCFDA reagent for 30 min, and the ROS generation was determined by flow cytometry. Bar chart indicates the ROS levels in A549 and H1299 cells; bars, S.D. * *p* ≤ 0.05; *** *p* ≤ 0.001. A549 cells (**C**) and H1299 cells (**D**) were treated with PPVI or co-treated with PPVI and NAC for 24 h. The cells were then incubated with 5 µM H2DCFDA reagent for 30 min, and the ROS generation was determined by flow cytometry. Bar chart indicates the ROS levels in A549 and H1299 cells; bars, S.D. * *p* ≤ 0.05; ** *p* ≤ 0.01; *** *p* ≤ 0.001.

As is known to us, cancer cells die in three main ways—apoptosis, autophagy and pyroptosis/necrosis [34]—and excessive ROS, mainly generated from the damaged mitochondrial, has been proven to a big trigger of apoptosis, autophagy and pyroptosis [35]. Thus, we here aimed to investigate whether PPVI can increase ROS levels in A549 and H1299 cells by employing H2DCF-DA as a fluorescence probe together with flow cytometry analysis for ROS measurement. As shown in Figure 7A,B, PPVI significantly increased the intracellular ROS level in A549 and H1299 cells. Furthermore, NAC, as the classical ROS scavenger, could remarkably decrease the ROS level in PPVI-treated A549 and H1299 cells (Figure 7C,D). Therefore, these data suggest that PPVI could increase the intracellular ROS level in A549 and H1299 cells.

### 2.6. PPVI Activates ROS-Triggered NF-κB Signaling Pathway in A549 and H1299 Cells

**Figure 8:**
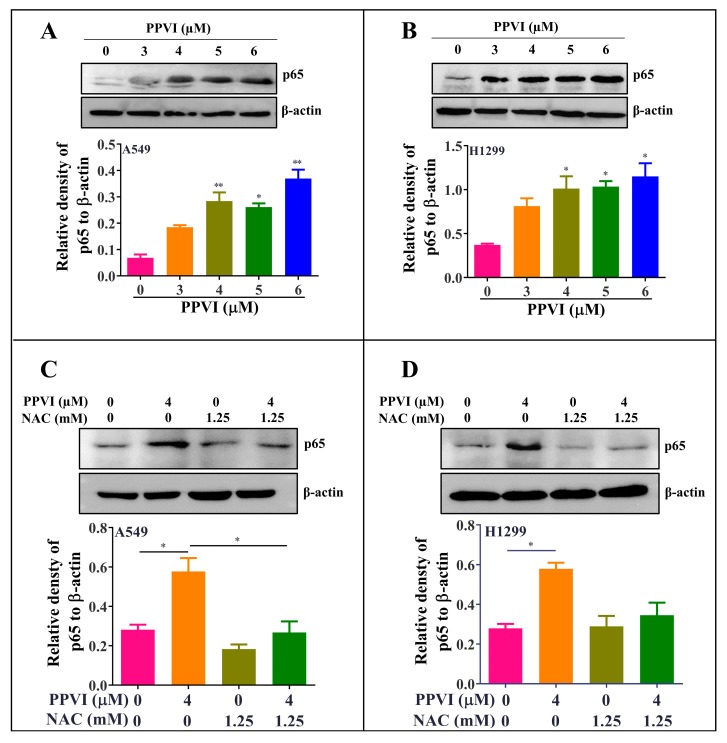
PPVI activated the ROS/NF-кB pathway in A549 and H1299 cells. A549 cells (**A**) and H1299 cells (**B**) were treated with PPVI as indicated by the graded concentrations for 24 h. A549 cells (**C**) and H1299 cells (**D**) were treated with PPVI or co-treated with NAC and PPVI, as indicated by the concentration shown, for 24 h. After treatment, cell protein was then harvested to detect p65 and β-actin by Western blot. Bar chart indicates the relative density of p65 to β-actin; bars, S.D. *p* ≤ 0.05; * *p* ≤ 0.01. The full-length Western blotting images are shown in Figure S6.

Growing evidence shows that ROS, as a trigger, promotes the release of pro-inflammatory cytokines as well as the activation of pro-inflammatory transcription factors (NF-κB) [36]. Furthermore, the NF-κB pathway was primarily thought to modulate the activation of the NLRP3 inflammasome and the gene expression of NLRP3 [37]. In this study, the protein expression of p65 (NF-κB) in PPVI-treated A549 and H1299 cells was firstly determined by using Western blot. As shown in Figure 8A,B, PPVI dose-dependently increased p65 protein expression. To investigate whether the increased ROS level participates in the activation of NF-κB in PPVI-treated A549 and H1299 cells, the protein expression of p65 in PPVI or PPVI plus NAC treated A549 and H1299 cells was detected by using Western blot. As we expected, NAC significantly inhibited PPVI-induced p65 protein expression (Figure 8C,D). Therefore, concluded that PPVI activated NF-κB signaling pathway via increasing the intracellular ROS level in A549 and H1299 cells.

### 2.7. PPVI Induces Pyroptotic Cell Death via the ROS/NF-κB Pathway in A549 and H1299 Cells

**Figure 9:**
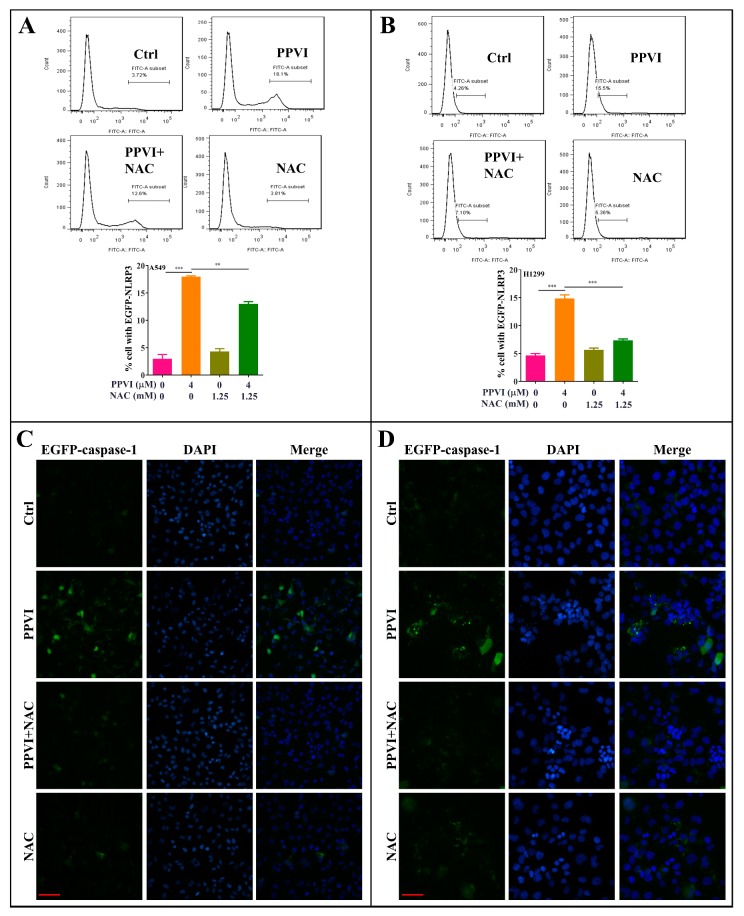
NAC inhibits the expression of NLRP3 and caspase-1 in PPVI treated A549 cells and H1299 cells. A549 cells (**A**) and H1299 cells (**B**) transiently transfected with EGFP-N1-NLRP3 plasmid for 24 h, treated with PPVI or co-treated with NAC and PPVI, at indicated concentrations, for another 24 h. After treatment, the cells were trypsinzed and collected for the analysis of fluorescence intensity by flow cytometry. Bar chart indicates the percentage of cells with GFP signal in A549 and H1299 cells; bars, S.D. ** *p* ≤ 0.01; *** *p* ≤ 0.001. A549 cells (**C**) and H1299 cells (**D**) transiently transfected with EGFP-N1-caspase-1 for 24 h were treated with PPVI or co-treated with NAC and PPVI at indicated concentrations for another 24 h. After treatment, the cells were fixed with 4% PFA, and the representative images were captured using the fluorescence microscope. Magnification: 40×. Scale bar: 25 μm.

**Figure 10:**
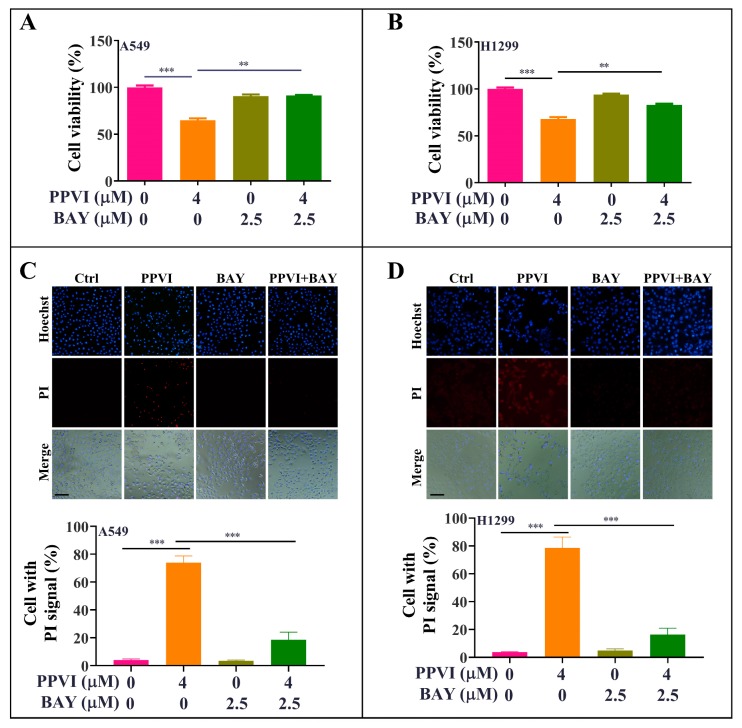
BAY-inhibited, PPVI-induced pyroptotic cell death in A549 cells and H1299 cells. A549 cells (**A**) and H1299 cells (**B**) treated with PPVI or co-treated with PPVI and BAY at the indicated concentration for 24 h. After treatment, the cytotoxicity was measured by MTT method, and the bar chart indicates the cell viability of A549 and H1299 cells; bars, S.D. ** *p* ≤ 0.01; *** *p* ≤ 0.001. A549 cells (**C**) and H1299 cells (**D**) treated with PPVI or co-treated with PPVI and BAY at the indicated concentration for 24 h. The cells were then stained with 2 μg/mL PI (red, staining dying cells) plus 2 μg/mL Hoechst 33342 (blue, staining all cells) for 10 min, and then observed by fluorescent microscopy. Bar chart indicates the percentage of cells with PI signal compared to blue signal; bars, S.D. *** *p* ≤ 0.001. Magnification: 10×, Scale bar: 100 µm.

To investigate whether the increased ROS level is closely associated with the activation of the NLRP3 inflammasome and the corresponding induced pyroptotic cell death, we firstly determined the EGFP-NLRP3 expression by measuring the intensity of the GFP signal in transiently transfected A549 and H1299 cells by flow cytometry. As shown in Figure 9A,B, we found that NAC significantly decreased the GFP intensity of PPVI-treated A549 and H1299 cells. In addition, we have also observed the EGFP-caspase-1 expression in transiently transfected A549 and H1299 cells by fluorescence microscope. The Figure 9C,D displayed that the expression of caspase-1 was significantly inhibited by NAC in PPVI-treated A549 and H1299 cells. Therefore, these data suggest that the activation of the NLRP3 inflammasome was closely linked with ROS generation. Furthermore, by employing the MTT method, the cell death of A549 and H1299 cells, induced by PPVI, was remarkably reversed by NAC (Figure S3). Moreover, the PPVI-induced cell death of A549 and H1299 cells was reversed by the specific inhibitor of NF-κB, named Bay 11-7085 (BAY) (Figure 10A,B). The double Hoechst/PI staining results also displayed that BAY significantly decreased the PI uptake, which was increased by PPVI in A549 and H1299 cells (Figure 10C,D). Taken together, these data suggest that PPVI-induced pyroptosis in A549 and H1299 cells via ROS triggered NF-κB activation.

## 3. Discussion

NSCLC is a type of epithelial lung cancer that accounts for about 85% of all lung cancers [38]. As NSCLC is relatively insensitive to chemotherapy as compared to small cell carcinoma, more than one kind of treatment is commonly used, according to the stage of NSCLC. Emerging evidences show the combinational use of chemotherapeutic reagents has been applied to increase the five year survival rate of NSCLC [39]. Therefore, finding novel therapeutic strategies is urgent for NSCLC. Apoptosis, autophagy and pyroptosis are three main types of cell death [40]. Many traditional chemotherapies for NSCLC, such as gefitinib, cisplatin, paclitaxel, erlotinib and crizotinib, were reported to induce cell apoptotic and autophagic death to inhibit tumor growth and cell proliferation [41,42,43,44]. However, there are limited studies on the involvement of pyroptosis and related drugs for NSCLC.

Pyroptosis is an inflammatory of programmed cell death which occurs most frequently under the conditions of infection and antimicrobial response [7]. Emerging evidences indicate that inflammation plays an important role in the tumor development, including initiation, growth, invasion and metastasis [45]. Although there are many studies on the toll-like receptors (TLRs) or interferon (IFN) pathways in tumor development, the role of inflammasomes in tumors is poorly clarified [46]. Inflammasome is an innate immune pathway which is involved in the release of pro-inflammatory cytokines, such as IL-1β and IL-18. To date, various inflammasomes, including NLRP1, NLRP2, NLRP3, absent in melanoma (AIM2) and NLRC4, have been identified [47]. Among them, the NLRP3 inflammasome is the most well studied inflammasome [47]. Upon upregulation of the NLRP3 protein, caspase-1 begin as an inactive precursor called zymogen. The caspase-1 enzymes become activated when they oligomerize and form tetramers [47,48]. This cysteine-cleaved caspase-1 not only causes cell death but also cleaves the pro-inflammatory cytokines such as IL-1β and IL-18, and GSDMD [32,49]. In caspase-1-deficient mice, tumorigenesis was increased in the Azoxymethane–Dextran sodium sulfate (AOM–DSS)-induced, colitis-associated colon cancer model, and the colonic epithelial cell proliferation in the early stage of tumorigenesis, and tumor cell proliferation was found [50]. In addition, a cytochrome P450 1B1 inhibitor could suppresses the tumorigenicity of prostate cancer via the upregulation of caspase-1 [51]. Furthermore, some chemicals were reported to suppress the proliferation of NSCLC cells via the activation of pyroptosis. For example, simvastatin suppresses proliferation and migration in vitro and in vivo by inducing pyroptosis via activating NLRP3-caspase-1-IL-1β and IL-18 pathways [31]. In addition, cisplatin was reported to induce higher levels of secondary necrosis/pyroptosis in A549 cells via the caspase-3/GSDME activation [52]. Therefore, the activation of caspase-1 plays an important role in inhibiting the proliferation of cancer cells, and the compounds with an activating NLRP3 inflammasome effect have the potential to be the treatments for NSCLC. 

To date, many natural products from traditional Chinese medicines (TCMs) have been identified to have a potent anti-tumor effect [28,53]. TTM, a traditional Chinese medicine, was found to significantly suppress the proliferation of cancer cells and growth of tumors. It has been previously reported that PPVI, the saponin isolated from TTM, could induce cell cycle arrest and apoptosis by increasing p-p53 expression to inhibit the expression of cyclin B1 in A549 cells. Alternatively, PPVI activated p21 Waf1/Cip1 to inhibit the expression of cyclin B1 in H1299 cells. As is known to us, there is expression of p53 in A549 but none in H1299 cells. Therefore, the different effects of PPVI on the activation of the apoptosis pathway may due to the difference of the expression of p53 in A549 and H1299 cells [54]. In addition, we have recently reported that PPVI could significantly suppress the proliferation of NSCLC via the induction of apoptosis and autophagy in vitro and in vivo, which was regulated by the increased ROS levels and its resultant downregulation of the mTOR-signaling pathway [29]. Further study in the current animal experiment found that the NLRP3 inflammasome was activated in PPVI-administrated, A549-bearing athymic nude mice. Recent evidences suggested that multiple signaling pathways and various types of cell death may be activated in single dying cells [55,56], and ROS, as a trigger, can lead to various types of regulated cell death (RCD) [55,57,58]. Furthermore, these RCD share some common mechanisms [59], and many compounds, such as simvastatin, paclitaxel, cisplatin, etc., were reported to induce autophagy, apoptosis and pyroptosis in NSCLC [31,52,60,61,62,63,64]. Therefore, we suspected that PPVI might also induce pyroptotic cell death in NSCLC via increased ROS levels. In this study, we further confirmed that the effective concentrations (3–6 μM) of PPVI could increase ROS levels in A549 and H1299 cells. Emerging evidence indicates that the characteristic changes in the cell morphology of various cell death types are vacuolated cytoplasm and intact cell membrane for autophagy, cell rounding and shrinkage for apoptosis, and the appearance of membrane bubbles in a single big one for pyroptosis [12,58,59,65]. In order to observe the pyroptotic, autophagic and apoptotic cell deaths of NSCLC, we employed an ImageXpress Micro 4 Widefield High-Content Imaging System to monitor the dynamic change in the cell morphology of A549 cells treated with 6 μM of PPVI; the highest effective concentration was found to display a potent autophagic and apoptotic effect in NSCLC. The results shown in Figure 2C and Video S1 suggest that A549 cells became round and shrank at 3 h, and big membrane bubbles (indicated with red arrow) appeared at 6 h, which suggested that both a longer treatment time than cells treated with equal effective concentrations of PPVI, and a higher effective concentration of PPVI than cells treated with the same treatment time, were required for the induction of pyroptosis, as compared to the activation of autophagy and apoptosis in NSCLC. As already known, VX-765 is a specific inhibitor of caspase-1 and caspase-4, but not caspase-3, and also inhibits lipopolysaccharides (LPS)-induced IL-1β and IL-18 production in primary human peripheral blood mononuclear cell (PBMC) cultures [66]. Among them, IL-1β and IL-18 have been commonly considered as the markers of the induction of pyroptosis [67]. In this study, the cell death of A549 and H1299 cells induced by PPVI was decreased by the addition of VX-765 and NSA, which suggested that PPVI not only induces apoptosis but also induces pyroptosis via the cleavage of caspase-1. By determining the activation of NLRP3 inflammasome through the measurement of the expression of NLRP3, ASC, and caspase-1 using Western blot and immunofluorescence methods, we found that PPVI could significantly activate the NLRP3 inflammasome. Meanwhile, the pro-inflammatory cytokines, such as IL-1β and IL-18 and GSDMD, were cleaved, which ultimately induced pyroptotic cell death in A549 and H1299 cells.

**Figure 11:**
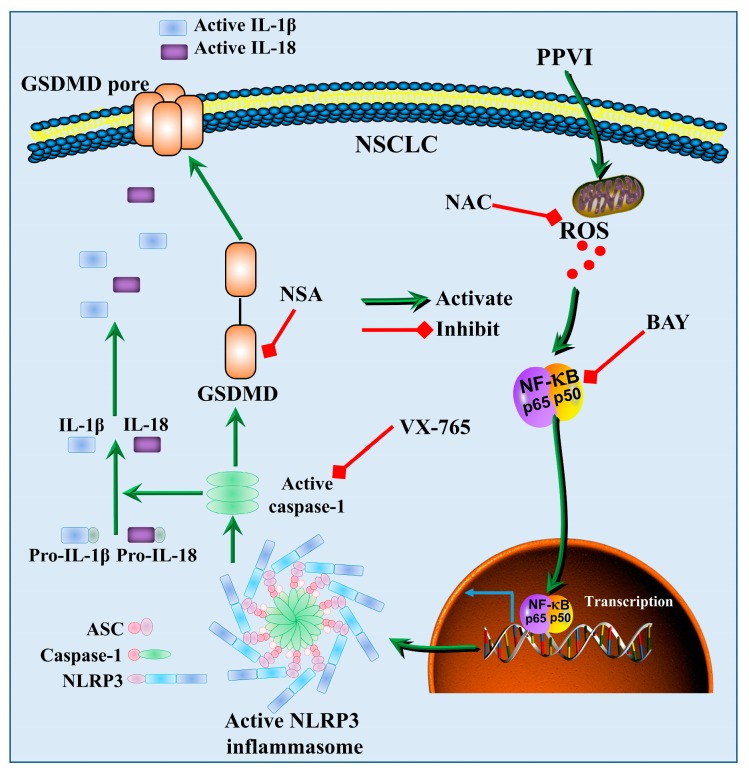
A schematic diagram of the molecular mechanism by which PPVI induced pyroptotic cell death via the activation of the ROS/NF-κB/NLRP3/GSDMD signal axis in A549 and H1299 cells.

Emerging evidence indicates that the NLRP3 inflammasome is activated by a series of endogenous materials such as adenosine triphosphate, uric acid, and number of exogenous agents, including bacterial hemolysins, silica, asbestos, uric acid and alum [68]. Recently, the increased cellular generation of ROS has been found in response to the above activators. For example, crystalline silica activates the NLRP3 inflammasome through ROS and the caspase-1-dependent pathway [69]. In addition, ROS induced by silica was decreased in NLRP3-deficient macrophages [70]. Therefore, ROS is an upstream event of NLRP3 inflammasome activation. In addition, NF-κB proteins, a family of transcription factors, play an important role in inflammation and immunity [71]. Recent studies suggest that the NF-κB pathway was recognized to regulate the activation of the NLRP3 inflammasome [72]. Moreover, ROS could activate NF-κB through the IκB kinase (IKK)-dependent pathway [73]. Therefore, the ROS/NF-κB signaling pathway plays an important role in the activation of the NLRP3 inflammasome. For the NF-κB signaling pathway in the NLRP3 inflammasome, most researchers found that Acetyl-NF-κB p65 (Lys310) was upregulated and the NF-κB signaling pathway was activated, and ultimately activated the NLRP3 inflammasome [74,75,76,77]. Therefore, the Acetyl-NF-κB p65 (Lys310) antibody, which recognizes the overexpressed levels of the NF-κB p65 protein acetylated at Lys310, was also used in this study. The result showed that PPVI significantly increases ROS level and activates the NF-κB signaling pathway in A549 and H1299 cells. As expected, NAC could decrease PPVI-induced ROS generation. Furthermore, NAC also inhibited the NF-κB signaling pathway in PPVI-treated A549 and H1299 cells. To further validate the correlation of cell death with the activated NLRP3 inflammasome, as well as the increased ROS level in PPVI-treated A549 and H1299 cells, we found that NAC could reverse NLRP3 expression and cell death induced by PPVI. Moreover, BAY could increase the cell viability of PPVI-treated A549 and H1299 cells. Therefore, all the data in this study were summarized in the diagram showed in Figure 11, which clearly clarified the anti-proliferation effect of PPVI and its action mechanism in NSCLC. In conclusion, our study showed for the first time that PPVI isolated from TTM induces caspase-1-mediated pyroptotic cell death via the activation of the ROS/NF-κB/NLRP3/GSDMD signal axis in A549 and H1299 cells. These findings provide evidence and novel insights for PPVI’s development into a novel candidate for the treatment of NSCLC.

## 4. Materials and Methods 

### 4.1. Reagents, Antibodies and Plasmids

Polyphyllin VI (PPVI, purity > 98%) was previously isolated from TTM by us. Belnacasan (VX-765, T6090) and necrosulfonamide (NSA, T6904) were purchased from Topscience Co., Ltd. (Shanghai, China). *N*-acetyl-L-cysteine (NAC, A7250), 3-(4,5-dimethylthiazol-2-yl)-2,5-dimethyltetrazolium bromide (MTT, M2128), Hoechst 33342 (B2261) and propidium iodide (PI, P4170) were bought from Sigma (St. Louis, MO, USA). Annexin V-FITC/PI Apoptotic detection kit was purchased from Vazyme Biotech Co., Ltd. (Nanjing, China). Caspase-1 Activity Assay Kit (Colorimetric) was purchased from Abbkine Inc., (Wuhan, China, #KTA3020). LIVE/DEADTM Cell Imaging Kit (488/570) was obtained from Invitrogen (Carlsbad, CA, USA). Milli-Q water was prepared by Milli-Q integral water purification system (Millipore, Billerica, MA, USA) in our laboratory. The primary antibodies used in the current study were as follows: Acetyl-NF-κB p65 (Lys310) (12629), IL-1β (12242) and NLRP3 (15101) from Cell Signaling Technology Inc, (CST, Beverly, MA, USA); Caspase-1 (A1115) and IL-18 (A0964) from ABclonal Biotechnology Co., Ltd. (Wuhan, China); β-actin (M177-3) from MBL International (Woburn, MA, USA); ASC (sc-514415) from Santa Cruz Biotechnology Inc., (Texas, USA); GSDMD from Proteintech Group, Inc., (Wuhan, China). Human IL-1β ELISA Kit (CHE0001) and human IL-18 ELISA Kit (CHE0007) were purchased from 4A Biotech Co., Ltd., (Beijing, China). The plasmids including pmCherry-C1-ASC (PPL01752-2b), pEGFP-N1-NLRP3 (PPL00151-2a) and pEGFP-N1-caspase-1 (PPL00392-2e) were bought from Public Protein/Plasmid Library (PPL, Nanjing, China).

### 4.2. Animal and Drug Administration

In this study, all the animal experiments were approved with a No. 201903-143 by the Animal Ethics Committee (AEC) of the Southwest Medical University, and performed as described previously [29]. Briefly, six week old female and male athymic nude mice purchased from Chengdu Dashuo Experimental Animal Co., Ltd. (Chengdou, China) were maintained in pathogen-free conditions. A549 cells (1 × 10^6^/mouse in 0.1 mL culture medium) were injected subcutaneously into the right flank of the nude mice. After 10 d, the mice were randomly divided into five groups (eight mice per group) as follows: control group (0.9% NaCl), PPVI-treated groups (2.5, 5, and 10 mg/kg) and gefitinib (20 mg/kg, positive group). After intraperitoneal (I.P.) administration for 10 consecutive days, the mice were sacrificed and tumor tissue was collected for the detection of activation of the NLRP3 inflammasome by Western blotting and immunohistochemistry methods.

### 4.3. Immunohistochemistry

The measurement of the NLRP3 inflammasome on tumors was performed by measuring the expression of NLRP3, IL-1β and GSDMD using immunohistochemistry method. In brief, paraffin sections were dewaxed in xylene and washed two times, then rehydrated by 100%–60% ethanol (100%–60%) and distilled in water three times. After adding the citric acid repair solution, the slice was placed in a microwave oven and fixed once for 15 minutes. After that, it was cooled in the repair solution, and then was rinsed with phosphate-buffered saline (PBS) three times for one minute each time. After washing, 3% H_2_O_2_ was added and the slice was rinsed with PBS three times at room temperature for 1 h. The sections were blocked with 5% blocking serum for 1 h at room temperature, and then incubated with 50 uL of primary antibody including NLRP3, IL-1β or GSDMD at 4 °C overnight. On the second day, 50 μL of anti-rabbit/mouse horseradish peroxidase (HRP)-labeled polymer was added and followed by an incubation for 2 h at 37 °C. Then, the pre-formed developer diaminobenzidine (DAB) working solution was added and continued to incubate for three minutes at room temperature, and immediately rinsed with distilled water. After that, the slice was hematoxylin counterstained for one minute, and then rinsed with distilled water. Finally, the slice was dehydrated, sealed and mounted with neutral gum. The representative images were captured under the fluorescence microscope (Nikon ECLIPSE 80i, Tokyo, Japan).

### 4.4. Cell Culture

The NSCLC wild-type (WT) EGFR cell lines (A549 and H1299), and EGFR-mutated cell line (PC-9) were purchased from Conservation Genetics Chinese Academy of Sciences Kunming Cell Bank (Chinese Academy of Medical Sciences, Kunming, China). Cells were cultured in Roswell Park Memorial Institute 1640 (RPMI 1640) or Dulbecco’s modified Eagle’s medium (DMEM) supplemented with 10% (vol/vol) fetal bovine serum (FBS), 50 U/mL penicillin and 50 mg/mL streptomycin (Invitrogen, Scotland, United Kingdom). All cells were grown at 37 °C in a 5% CO_2_ humidified incubator.

### 4.5. MTTAssay

The cytotoxicity of PPVI in A549 and H1299 cells was measured by MTT assay. In brief, A549 or H1299 cells seeded onto 96-well cell plates at a density of 5 × 10^3^ cells/well were treated with PPVI under the indicated concentrations for 24, 48 and 72 h. After treatment, 10 µL of MTT solution (5 mg/mL) was added to each well and continued to incubate for another 4 h. The medium was then removed and 150 µL of DMSO was added into each well to dissolve the formed formazan in cells. After shaking at low speed for 10 min at room temperature, the colorimetric reading of the solute mixture was then determined by spectrophotometer (BioTek, VT Lab, USA) at OD 570 nm. The cytotoxicity was determined by calculating cell viability percentage using the following formula: Cell viability (%) = Cells _number treated_/Cells _number control_ × 100.

### 4.6. Lytic Cell Death Assay

The lytic cell death of A549 and H1299 cells was measured by PI incorporation, as described previously [78]. In brief, cells were seeded in 24-well plates and treated with indicated concentrations of PPVI. After treatment, cells were stained by Hoechst 33342 solution (5 μg/mL) to reveal cell nuclei, and PI solution (2 μg/mL) to indicate necrotic cells according to the manufacturer’s instructions. The representative images with blue or red fluorescence signal at the same field were captured using the fluorescence microscope (Nikon ECLIPSE 80i, Tokyo, Japan) with 10 × magnification. The percentage of cell with PI signal were then calculated using Image J software (National Institutes of Health, Bethesda, MD, USA).

The dynamic change in cell morphology and the cell death process of A549 cells was performed by real-time observation using ImageXpress Micro 4 Widefield High-Content Imaging System (Molecular Devices, San Jose, CA, USA). Briefly, A549 cells seeded on 6-well plate were stained with Hoechst 33342 solution (5 μg/mL) and PI solution (2 μg/mL), which was diluted with RPMI 1640, and treated with 10 μM PPVI for 4 h. The real-time images of A549 cells which were treated with PPVI from 0–4 h were captured. Finally, a 10 s video that records the cell morphology of A549 was obtained.

### 4.7. Flow Cytometry Analysis

As is known to us, Annexin-V recognizes phosphatidylserine exposed on the external leaflet of the plasma membrane of the apoptotic cells, and it also can stain cells undergoing pyroptosis due to the membrane rupture that allows for the recognition of phosphatidylserine on the inner leaflet [79]. Therefore, the Annexin V-FITC/PI detection kit, widely used to measure cell apoptosis, was also used to detect pyroptosis in lots of studies of non-small lung cancer [80,81,82]. In our study, pyroptosis was measured by the annexin V-FITC/PI Detection Kit from BD Biosciences (San Jose, CA, United States), and the pyroptotic (PI positive) and apoptotic (Annexin V-FITC positive) cells could be successfully analyzed and obtained [79,80]. Briefly, A549 and H1299 cells were treated with PPVI at indicated concentrations for 24 h. After treatment, cells were harvested, washed with PBS twice and stained using the Annexin V-FITC/PI Apoptosis assay kit by following the manufacturer’s instructions. After incubation at room temperature for 15 min in the dark, the stained cells were analyzed on the FACSVerse flow cytometer (BD Biosciences, San Jose, CA, USA). Data acquisition and analysis were performed using the Flowjo software (BD Biosciences, San Jose, CA, USA).

### 4.8. Caspase-1 Activity Assay

Caspase-1 activity in A549 cells and H1299 cells was measured using a commercial Caspase-1 Activity Assay Kit (Colorimetric) according to the manufacturer’s instructions. This assay is performed on the basis of the ability of caspase-1 to change acetyl-Tyr-Val-Ala-Asp p-nitroaniline (Ac-YVAD-pNA) into the yellow formazan product pNA. After treatment, cells were harvested and lysed. Protein concentration was then determined by using the Bradford protein assay reagent (Bio-Rad, CA, USA) according to the manufacture’s instructions. Absorbance was measured at 405 nm by spectrophotometer (BioTek, VT Lab, USA). Standard curves for the assay system were obtained from dilutions of the standards of pNA. Caspase-1 activity was then obtained by determining the amount of pNA according to the standard curve of pNA [83].

### 4.9. Transfection

To observe the activation of the NLRP3 inflammasome in cells, A549 or H1299 cells seeded on coverslips in 6-well plates were transfected with pEGFP-N1-NLRP3, pmCherry-C1-ASC or pEGFP-N1-caspase-1 plasmid using Exfect^®^ Transfection Reagent (Vazyme Biotech Co., Ltd., Nanjing, China) according to the manufacture’s instruction. After a 24 h of incubation, A549 or H1299 cells were then treated with PPVI under the indicated concentrations for 24 h. After treatment, cells were fixed with 4% PFA and stained with DAPI solution for 30 min. The cells were then analyzed using the fluorescence microscope (Nikon ECLIPSE 80i, Tokyo, Japan) with 10× magnification and the representative images were captured. The fluorescence intensity, representing the expression of NLRP3, ASC and the cleavage of caspase-1, was quantified using ImageJ software (National Institutes of Health, Bethesda, MD, USA).

### 4.10. Western Blot

After treatment, cells or tumor tissue were lysed with 1× RIPA lysis buffer (CST, MA, USA) containing a protease inhibitor cocktail. The lysate was centrifugated and the supernatant was transferred into a new tube. The protein concentration of the lysate was then determined by using the Bradford protein assay reagent (Bio-Rad, CA, USA) according to the manufacture’s instructions. An equal amount of the protein (30 μg per sample) was loaded onto SDS-PAGE for the protein separation. After electrophoresis, the protein on the SDS-PAGE was transferred to the polyvinylidene fluoride (PVDF) membrane, which was then blocked with 5% no-fat milk in TBST for 1 h at room temperature. After washing with TBST, the membrane was incubated with the primary antibodies overnight at 4 °C. On the second day, the membrane was washed with TBST three times and further incubated with HRP-conjugated secondary antibodies for 1 h at 37 °C. Finally, the bands on the membrane were revealed by UltraSignal Hypersensitive ECL Chemiluminescent Substrate (4A Biotech Co., Ltd., Beijing, China) and detected by the ChemiDoc MP Imaging System (Bio-Rad, California, USA). The band intensity of proteins was quantified by using ImageJ software (National Institutes of Health, Bethesda, MD, USA), and the relative expression of protein to β-actin was normalized and obtained.

### 4.11. ELISA

IL-1β and IL-18 levels in cell supernatant of the PPVI-treated A549 cells and H1299 cells were detemined by using a human IL-1β ELISA kit and a human IL-18 ELISA kit according to the manufacturer’s instructions. In brief, after treatment, the culture medium was collected for centrifugation at a speed of 1000× *g* for 10 min. The supernatants were analyzed by ELISA kit to detect the cytokines, including IL-1β and IL-18. Absorbance was measured at 450 nm by spectrophotometer (BioTek, VT Lab, USA). Standard curves for the assay system were obtained from dilutions of the standards of IL-1β and IL-18 ELISA kit; the concentrations of IL-1β and IL-18 were then obtained by extrapolation from the standard curve [84].

### 4.12. LIVE/DEAD Cell Imaging

Cell death of A549 and H1299 cells was also analyzed and observed using the LIVE/DEAD Cell Imaging kit according to the manufacturer’s instructions. Briefly, after treatment, the medium was removed and the cells were stained with the mixture of green dye and red dye solution, diluted with culture medium, for 20 min. After staining, the representative images with red or green fluorescence signal were captured using the fluorescence microscope (Nikon ECLIPSE 80i, Japan) with 10 x magnification. The images at the same field were merged and analyzed using ImageJ software (National Institutes of Health, Bethesda, MD, USA). Cell death was determined through the calculation of the ratio of cells in red to total cells, and a minimum of 1000 cells from each sample were scored by three randomly selected fields.

### 4.13. ROS Level Measurement

In the current study, the intracellular ROS level was determined as previously described by flow cytometry analysis using H2DCF-DA fluorescence probe [27,85]. Briefly, after PPVI treatment, A549 or H1299 cells were trypsinzed and collected in 1.5 mL tubes. The cell suspension was then centrifugated at a speed of 2000 rpm for 5 min. After that, the supernatant was removed and the remained cell pellet was then resuspended in 5 µM H2DCF-DA, diluted with culture medium, and continued to incubate for 30 min in the dark. After incubation, the cell pellet was washed twice with PBS and resuspended with 500 µL of PBS. The cell suspension was then subjected to flow cytometry analysis for measurement of ROS level on a FACSVerse flow cytometer (BD Biosciences, San Jose, CA, USA). Data acquisition and analysis were performed by the Flowjo software (BD Biosciences, San Jose, CA, USA).

### 4.14. Statistical Analysis

Statistical difference between groups was analyzed by using one-way analysis of variance (ANOVA) followed by Tukey’s multiple-comparison post hoc test or Dunnett. All data presented as mean ± SD were analyzed using Graph Prism 5.0 software (San Diego, CA, USA). *p* < 0.05 was considered to have statistical significance.

## 5. Conclusions

This study further found that PPVI could activate the NLRP3 inflammasome in A549-bearing athymic nude mice. Moreover, we revealed that PPVI induced an apoptosis-to-pyroptosis switch, and ultimately cell death, in A549 and H1299 cells via the activation of caspase-1, which was closely associated with the ROS/NF-κB/NLRP3/GSDMD signal axis. Therefore, we concluded that PPVI could also inhibit the progress of NSCLC via the pyroptosis, apart from both autophagy and apoptosis, and therefore PPVI might be valuable for the further development of a new candidate for the future treatment of NSCLC.

## Supplementary Materials

The following are available online at https://www.mdpi.com/2072-6694/12/1/193/s1, Figure S1: The cytotoxicity of PPVI in A549 cells (A) and H1299 cells (B) for 24, 48 and 72 h treatments, and in A549 cells, H1299 cells and PC-9 cells for 48 h (C) were measured by using MTT assay, Figure S2: A549 cells (A) and H1299 (B) were treated with PPVI or co-treated with PPVI and VX-765 under the indicated concentrations for 24 h. After treatment, cells were then stained with LIVE/DEAD staining solution for 15 min. Representative images were captured by fluorescence microscope. Bar chart indicating cell death of A549 and H1299 cells. Magnification: 10×, Scale bar: 100 µm, Figure S3: A549 cells (E) and H1299 cells (F) treated with PPVI or co-treated with PPVI and NAC at the indicated concentration for 24 h. After treatment, the cytotoxicity was measured by the MTT method, and the bar chart indicates the cell viability of A549 and H1299 cells; bars, S.D. *** *p* ≤ 0.001, Figure S4: The full-length Western blotting images (1), Figure S5: The full-length Western blotting images (2), Figure S6: The full-length Western blotting images (3), Video S1: The time-lapse phase-contrast and fluorescent video of A549 cells stained with PI and Hoechst 33342 solution, taken at the indicated timepoints after the stimulation of PPVI.
